# The impact of ATP-sensitive potassium channel modulation on mitochondria in a Parkinson’s disease model using SH-SY5Y cells depends on their differentiation state

**DOI:** 10.1007/s10863-024-10018-x

**Published:** 2024-04-30

**Authors:** A Evinova, E Baranovicova, D Hajduchova, K Dibdiakova, I Baranova, P Racay, J Strnadel, R Pecova, E Halasova, M Pokusa

**Affiliations:** 1 Biomedical Centre Martin, Jessenius Faculty of Medicine, Comenius University, Bratislava, Slovakia; 2 Department of Pathological Physiology, Jessenius Faculty of Medicine, Comenius University, Bratislava, Slovakia; 3 Department of Medical Biochemistry, Jessenius Faculty of Medicine, Comenius University, Bratislava, Slovakia

**Keywords:** Potassium inward rectifying channel 6.2, Neurodegeneration, Mitochondria, SH-SY5Y, Glibenclamide, Diazoxide, Rotenone

## Abstract

Inward rectifying potassium channels sensitive to ATP levels (KATP) have been the subject of investigation for several decades. Modulators of KATP channels are well-established treatments for metabolic as well as cardiovascular diseases. Experimental studies have also shown the potential of KATP modulation in neurodegenerative disorders. However, to date, data regarding the effects of KATP antagonists/agonists in experiments related to neurodegeneration remain inconsistent. The main source of confusion in evaluating available data seems to be the choice of experimental models. The present study aims to provide a comprehensive understanding of the effects of both opening and blocking KATP channels in two forms of SH-SY5Y cells. Our results offer valuable insights into the significance of metabolic differences between differentiated and non-differentiated SH-SY5Y cells, particularly in the context of glibenclamide and diazoxide effects under normal conditions and during the initiation of pathological events simulating Parkinson’s disease in vitro. We emphasize the analysis of mitochondrial functions and changes in mitochondrial network morphology. The heightened protein expression of KATP channels identified in non-differentiated SH-SY5Y cells seems to be a platform for a more significant impact of KATP modulators in this cell type. The efficiency of rotenone treatment in inducing morphological changes in the mitochondrial network depends on the differentiation status of SH-SY5Y cells.

## Introduction

The regulation of potassium balance is crucial for maintaining homeostasis in the human body. A distinct subtype of potassium channels is sensitive to the energy status of the cell, known as ATP-sensitive potassium (KATP) channels. KATP acts specifically as the inwardly-rectifying potassium channels and is composed alternately from Kir 6.1 and Kir 6.2 subunits, which usually form homo- or hetero-tetramers depending on tissue specificity (Chan et al. 2008). In addition to transmembrane subunits rectifying K^+^ currents, each Kir monomer of ion channel is enriched by distinct type of sulfonylurea receptor (SUR) serving as a regulatory domain with high affinity to ATP, which has an antagonistic function on potassium permeability (Kuang et al. 2015; Kravenska et al. 2021). Kir 6.1 domains are adjusted to form a tandem with SUR 2A/B units, while Kir 6.2 shows higher affinity for SUR1 type of receptor (Ashcroft and Gribble 1998). KATP channels primarily allow the movement of potassium ions into the cell, but the fate of K^+^ is closely linked to the flow of Ca^2+^ ions across cell membranes, which is driven inwardly into the cytoplasm after the opening of voltage-gated calcium channels (Seino and Miki 2003). This phenomenon is particularly significant as it is influenced by KATP modulating agents used as a treatment. Glibenclamide, a synthetic KATP antagonist with an affinity for SUR1, is indicated in the treatment of diabetes mellitus II as it stimulates insulin secretion from pancreatic beta cells after calcium concentration increase in cytoplasm (Ashcroft and Rorsman 1989). Diazoxide, a potent Kir 6.2 agonist, is used to manage certain cases of hypertension (Vidt 1975) or hypoglycemia mainly by decreasing intracellular calcium levels (Grant et al. 1986). Kir 6.2, the product of the KCNJ11 gene, along with its selective modulators has become a subject of interest in research on neurodegenerative processes. This is also due to findings regarding its involvement in the regulation of cell death through its enhanced effect on mitochondria, where KATPs are also found (Ardehali and O’Rourke 2005). In addition to the Kir channels, a novel type of ATP dependent channel encoded by the CCDC51 gene was recently identified in mitochondria. Similar to Kir 6.2/SUR1, high affinity to diazoxide and glibenclamide was observed during study of this type of potassium channel (Paggio et al. 2019). Although the effects of potassium channels are undeniable in relation to changes in mitochondrial dynamics and respiratory activity (Liu et al. 2022; Peng et al. 2018), forming a clear opinion on the exact effects of specific KATP modulators on these processes remains challenging due to differences in experimental models.

KATP agonists have shown positive effects in animal and cell models, such as in ischemia-reperfusion injury (Akao et al. 2001; Nakagawa et al. 2013; Lei et al. 2018) neuroinflammation, and the formation of oxidative radicals (Rodríguez et al. 2013), as well as in Alzheimer’s and Parkinson’s type neurodegeneration (Ma and Chen 2004; Yang et al. 2005; Ma et al. 2009). Surprisingly, KATP antagonists, particularly glibenclamide, have also demonstrated positive effects on the symptoms of Parkinson’s disease (Abdelkader et al. 2020), Alzheimer’s disease in animal models (Esmaeili et al. 2020), and neuroinflammation (Jiang et al. 2021). These inconsistent findings highlight the lack of consensus in understanding the exact effects of KATP modulators in relevant experimental models of neurodegeneration. One hypothesis explaining the discrepancies across several studies could be attributed to the non-uniform distribution of Kir 6.2/6.1 throughout cell types. Kir 6.2 channels with affinity to SUR1 are preferentially expressed in organs such as the heart, or prostate and it is the most prevalent KATP forming unit in different regions of the brain (Fagerberg et al. 2014; Ashcroft and Gribble 1998). High density of KATP channels has been confirmed in hippocampal neurons, glial cells, astrocytes, as well as in neurons of the hypothalamus and substantia nigra (Seino and Miki 2003). This diversity may be a crucial consideration for potential future treatments.

The primary objective of the present study is to compare the effects of Kir 6.2/SUR1 modulators on mitochondrial functions, morphology and metabolism in two distinct forms of SH-SY5Y cell lines, which were prepared using different growth protocols. Human neuroblastoma-derived SH-SY5Y cells serve as a well-established model for the study of the mechanisms of neurodegeneration associated with Parkinson’s disease (Ioghen et al. 2023) due to their ability to differentiate into dopaminergic neurons, the primary type of neurons affected in Parkinson’s disease. Non-differentiated SH-SY5Y cells exhibit behavior similar to standard cancer cells, while differentiated SH-SY5Y cells manifest a typical neuronal phenotype with modulated mitochondrial activity more relevant to dopaminergic neurons (Forster et al. 2016). We are aware of a wide range of experimental studies dealing with both types of SH-SY5Y cells and the differences between them. However, we have noticed the missing correlation of up-to-date knowledge about the roles of the mostly studied KATP modulators (diazoxide and glibenclamide) in distinct experimental models and several contradicting findings. Our hypothesis is therefore set up to confirm the specificity of targeted cells during the propagation of KATP modulators effects as a source of documented discrepancies. To address this point, we have induced patterns resembling neurodegeneration by mitochondrial complex I inhibition in both types of the studied cell lines. Our investigation aims to assess the impact of KATP (Kir 6.2/SUR1) modulators on selected parameters of energy metabolism and mitochondrial physiology in both control and pathological conditions after 24 hours of treatment.

## Material and methods

### Cell culture preparation

**Fig. 1:**
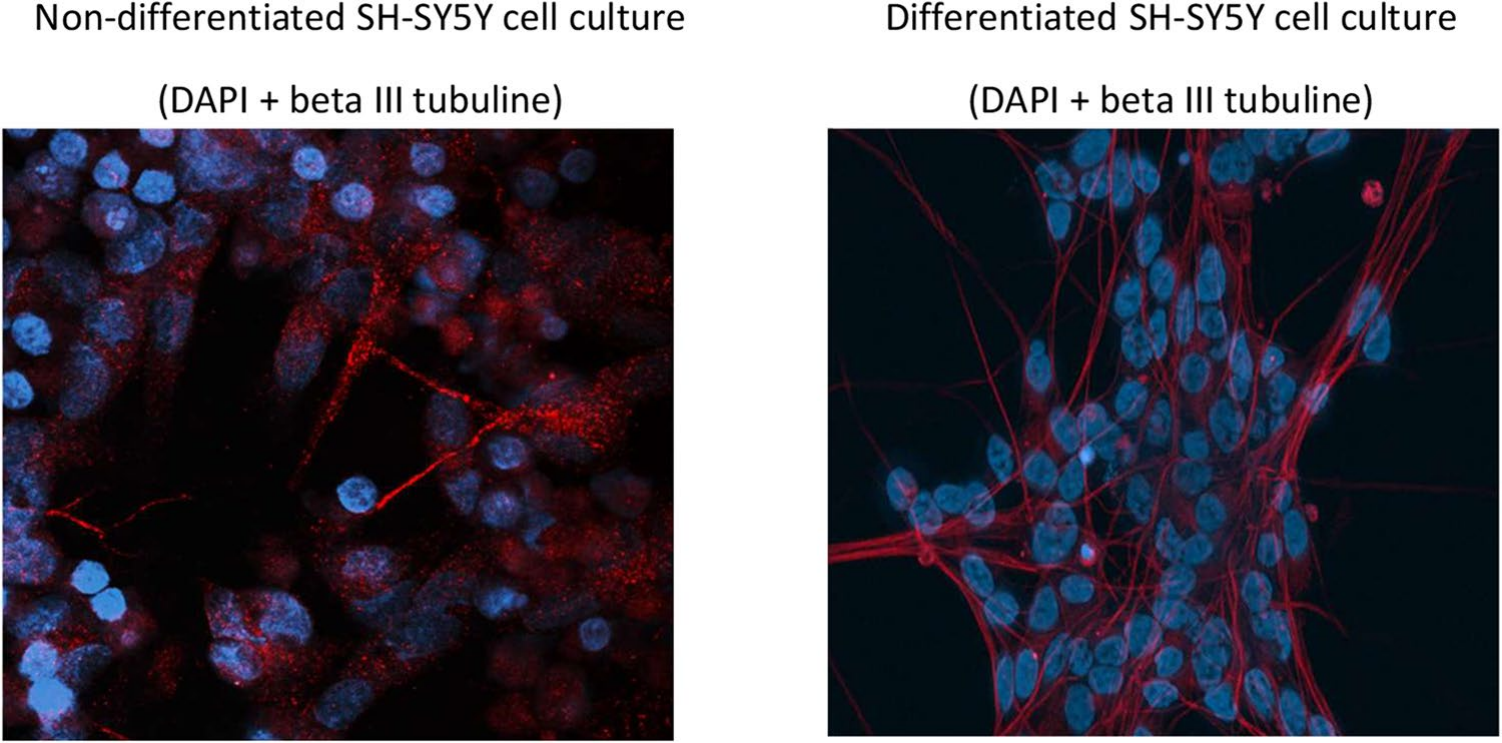
Phenotypic differences in SH-SY5Y cells based on growth protocol. Fluorescence images depict the distinction between differentiated and non-differentiated SH-SY5Y cell cultures. Nuclei are stained with DAPI (blue), and the beta III tubulin cytoskeleton is visualized using fluorescent antibodies (red)

A commercial SH-SY5Y cell line, obtained from the American Type Culture Collection, was employed for experimental procedures. Non-differentiated cell cultures were generated through standard procedures at 37 °C under a 5% CO_2_ humidified atmosphere, utilizing DMEM:F12 (Dulbecco’s Modified Eagle’s Medium and Ham’s F-12 Nutrient Mixture, Sigma) supplemented medium with 10% FBS and 1% penicillin streptomycin stock (all PAA). To induce differentiation in SH-SY5Y cells, an 18-day protocol involving 10 μM all-trans retinoic acid was implemented. This protocol was performed according to the original method established by Shipley et al. in 2016. Experimental groups for both cell types were prepared by subjecting them to a 24-hour treatment with either 20 μM glibenclamide (Merck) or 20 μM diazoxide (Merck), under control conditions or in the simultaneous presence of 50 nM rotenone (Sigma Aldrich). The phenotypic distinctions in SH-SY5Y cell cultures, cultivated according to distinct protocols, are illustrated in Fig.1.

### High-resolution respirometry

Mitochondrial respiration was assessed through high-resolution respirometry (HRR) measurements on intact cells, utilizing the two-chamber O_2_k-FluoRespirometer system (Oroboros Instruments), as previously detailed (Evinova et al. 2020; Brodnanova et al. 2021). Measurements were conducted on three biological replicates of both experimental and control cell cultures. For the HRR measurements, SH-SY5Y cells were suspended in the respiration medium MiR05-Kit and introduced into 2 ml glass chambers. The control coupling protocol for intact cells, involving the presence of oligomycin to inhibit mitochondrial ATP synthase, was employed to measure the leak respiration of the cells. Consistent with Oroboros protocols (Pesta and Gnaiger 2012), the resulting O2 flux values were employed to calculate basal respiration, maximal electron transport capacity, ATP-coupled respiration, spare respiratory capacity, and succinate-stimulated respiration. Residual oxygen consumption (ROX) was determined for flow correction in intact cell respiration.

### Western blot analysis

Proteins utilized for Western blot analysis were extracted from harvested cells employing RIPA lysis buffer supplemented with a 1x protease inhibitor cocktail (Halt™, Thermo Fisher). Thirty micrograms of proteins per lane were separated on 10% SDS-polyacrylamide gels under reducing conditions. Following electrophoretic separation, proteins were transferred to nitrocellulose membranes with a 0.2 μm pore size using a wet transfer method. The membranes were incubated for a minimum of 1 hour in a 2% BSA blocking solution and subsequently probed with antibodies specific to KCNJ11 (1:500, HPA048891, Sigma Aldrich). Following this, the membranes underwent incubation with secondary anti-rabbit antibodies (1:10,000, A9169, Sigma Aldrich). Immunopositive signals were visualized using the chemiluminescent substrate (GE Healthcare Amersham™ ECL) and the ChemiDoc XRS system (Bio-Rad). Western blot analysis was performed on two biological replicates and three technical replicates for each cell culture type. Non-differentiated SH-SY5Y cells served as the 100% control group.

### MTT test

SH-SY5Y cells were plated at a density of 10,000 cells per well in 96-well plates. The following day, cells were exposed to KATP modulators/rotenone for a duration of 24 hours. Control cells received the appropriate dose of DMSO as a vehicle. After a 20-hour incubation period, a 0.01 ml solution of MTT (Sigma Aldrich) at a concentration of 5 mg/ml was added to each well, and cells were further incubated for 4 hours. The insoluble formazan, generated through the reduction of MTT by viable cells, was dissolved by adding 0.1 ml of SDS solution (0.1 g/ml) followed by overnight incubation. The absorbance of formazan was measured spectrophotometrically using a Bio-Rad PR 2100 microplate reader. The relative viability of the cells was determined by comparing the optical density of formazan produced by treated cells to that of formazan produced by non-treated control cells. Results are graphically presented as a percentage of the control. Each experiment was conducted with three biological replicates of SH-SY5Y cell cultures. For each treatment time, the optical density value of non-treated control cells was considered as 100% viable cells.

### Confocal microscopy

The Zeiss LSM 880 scanning confocal microscope was utilized to compare the mitochondrial network and basal calcium concentration among the experimental groups of SH-SY5Y cell cultures. Staining with mitotracker Red FM or Fluo-4 probe (Thermo Fisher) was performed according to the manufacturer’s protocol, incorporating at least three biological replicates for each cell type and experimental group. A minimum of five microscopic images were acquired under identical acquisition parameters from each biological replicate using a wet objective with 40x magnification. Subsequently, the images were analysed for fluorescence intensity quantification. Mean intensity values were computed after correction for background fluorescence intensity. Evaluation of fluorescence signal intensity emitted by the mitotracker probe was used for the indirect quantification of inner mitochondrial potential. The fluo-4 fluorescent probe reflects calcium concentration through the quantification of fluorescence intensity. For the qualitative assessment of mitochondrial morphology changes, Image J software (FIJI) was employed, following the procedure outlined below.

### Calcium imaging

The experimental procedure employed an Inverted Zeiss Axio Observer D1 equipped with an MRm Axiocam and HXP 400 fluorescence light source. Real-time observation of changes in calcium homeostasis was conducted using the Fluo-4 vital fluorescence probe, known for its sensitivity to cytoplasmic calcium oscillations. For all real-time measurements of calcium changes, Tyrode’s solution (NaCl 130 mM, KCl 5 mM, CaCl_2_ 2 mM, MgCl 1 mM, Glucose 30 mM, Hepes 25 mM, pH 7.4) was utilized. Excitation of voltage-gated calcium channels was achieved by promptly replacing the standard Tyrode’s buffer with KCl Tyrode’s solution (NaCl 5 mM, KCl 130 mM, CaCl_2_ 2 mM, MgCl 1 mM, Glucose 30 mM, Hepes 25 mM, pH 7.4). Real-time measurements of the mean fluorescence intensity shift post-excitation were corrected for background fluorescence intensity. The fluorescence intensity during the basal condition before the potassium concentration increase was set as a zero level. The effect of each used experimental condition was evaluated by comparing the amplitudes of fluorescence increase after cell stimulation. Three biological replicates for both types of SH-SY5Y cell cultures were employed in total for data acquisition.

### Mitochondrial network analysis

**Fig. 2:**
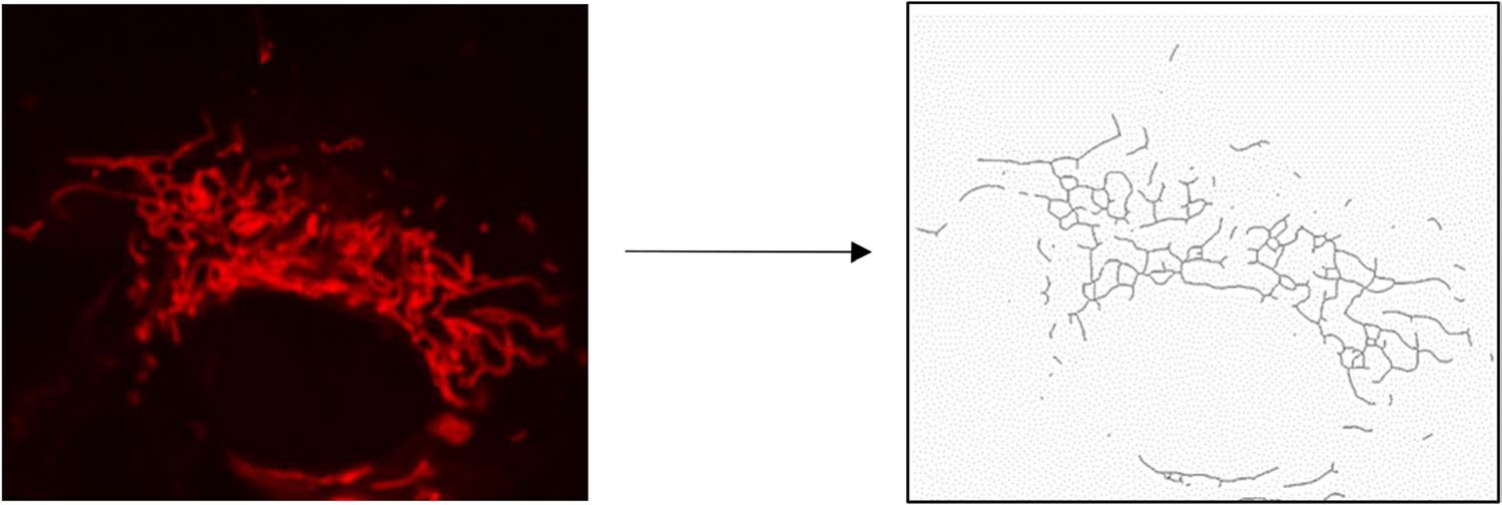
Schematic illustration of transformation used for obtained images of cellular mitochondrial network. The final binary image obtained from raw pictures of the mitochondrial network was proceeded to identification of voxel length, junction numbers and identification of solitary mitochondrial rod like structures

The analysis was conducted following a sequence of image processing steps based on the transformation of fluorescence images into binary 2D images (Fig. 2). Software protocol was outlined by Bakare et al. in 2021, utilizing Image J software. Parametrization of the mitochondrial network was performed to facilitate the identification of fragmentation, branching, and length pattern. For the characterization of the fragmentation status, the ratio between the counts of solitary rod-like mitochondria and the overall branches of mitochondria was calculated from the imaged cells. The branching pattern was characterized by the proportion of junctions counted against the total branch number observed in the cell. The average length of mitochondrial branches was determined by pixel overlap of separate mitochondrial branches under the same magnification and zoom conditions.

### Cell density evaluation

As supportive data to the outcomes from the MTT test, microscopical images obtained during confocal microscopy and calcium imaging were used for cell counts evaluation after used treatments. For the purpose of the analysis, the average cell counts from all images obtained from each experimental group served as a data output of one biological replicate. In total, five biological replicates of non-differentiated SH-SY5Y and three biological replicates of differentiated cells were imaged by confocal microscopy, while three biological replicates of both cell types were utilized for calcium imaging analysis.

### 1H-NMR spectra acquisition and analysis

The stock solution was composed of a phosphate buffer at 500 mM, adjusted to pH 7.4 (verified by pH meter reading), and approximately 0.2 mM TMS-d4 (trimethylsilylpropionic acid-d4) as a chemical shift reference, all dissolved in deuterated water. Subsequently, 500 μL of centrifuged cell media was meticulously mixed with 100 μL of the stock solution and transferred into a 5 mm NMR tube. Nuclear Magnetic Resonance (NMR) data were acquired using a 600 MHz NMR spectrometer, specifically the Avance III model from Bruker, equipped with a cryoprobe, operating at a temperature of 310 K. For evaluation, a modified Bruker CPMG profiling protocol was employed: FID size 64 k, dummy scans 4, number of scans 128, spectral width 20.0156 ppm, and a relaxation delay of 4 seconds. The multiplicity of peaks was confirmed in J-resolved spectra, and homonuclear cross peaks were verified in COSY spectra. Calculated peak integrals were utilized to determine the relative concentrations of metabolites in the cell medium collected after 24 hours of the relevant treatment (Table 1).

**Table 1:** Chemical shifts (in ppm), J couplings (in Hz) and multiplicities for the metabolites of interest as identified in cell medium by NMR. The proton NMR chemical shifts are reported relative to TMSP-d4 signal which was assigned a chemical shift of 0.000 ppm

Metabolite	NMR evaluation		NMR peak assignment, confirmed by jres and cosy
	from	to	
lactate	4.09	4.145	1.331(d; J = 6.97), 4.116(q; J = 6.97)
pyruvate	2.373	2.384	2.376(s)
glucose	3.885	3.906	3.233(m), 3.398(m), 3.458(m), 3.524(dd), 3.782(m), 3.824(m), 3.889(dd), 4.634(d), 5.233(d)

### Statistical analysis

Before the statistical evaluation of the obtained data, all data sets were tested for the presence of outliers in relevant experimental groups. Identified outliers were winsorized to the closest border values, which were defined as 1.5 times the interquartile range (IQR) below the first quartile (Q1) and 1.5 times the IQR above the third quartile (Q3). Data sets with non-normal distributions were further transformed using natural logarithm calculation. Normal distribution was then confirmed using the Shapiro-Wilk test. After normalizing the data distribution, a two-way ANOVA was employed to determine the statistical significance of the effects of rotenone and KATP modulators in the data obtained from the presented experiments. A significance level of p < 0.05 was set as the threshold for considering an effect as significant. In cases where a significant main effect was identified, a Tukey post hoc test was conducted for the relevant main factor. For the statistical evaluation of observed changes between non-differentiated and differentiated SH-SY5Y cells, an independent T-test was utilized.

## Results

**Fig. 3:**
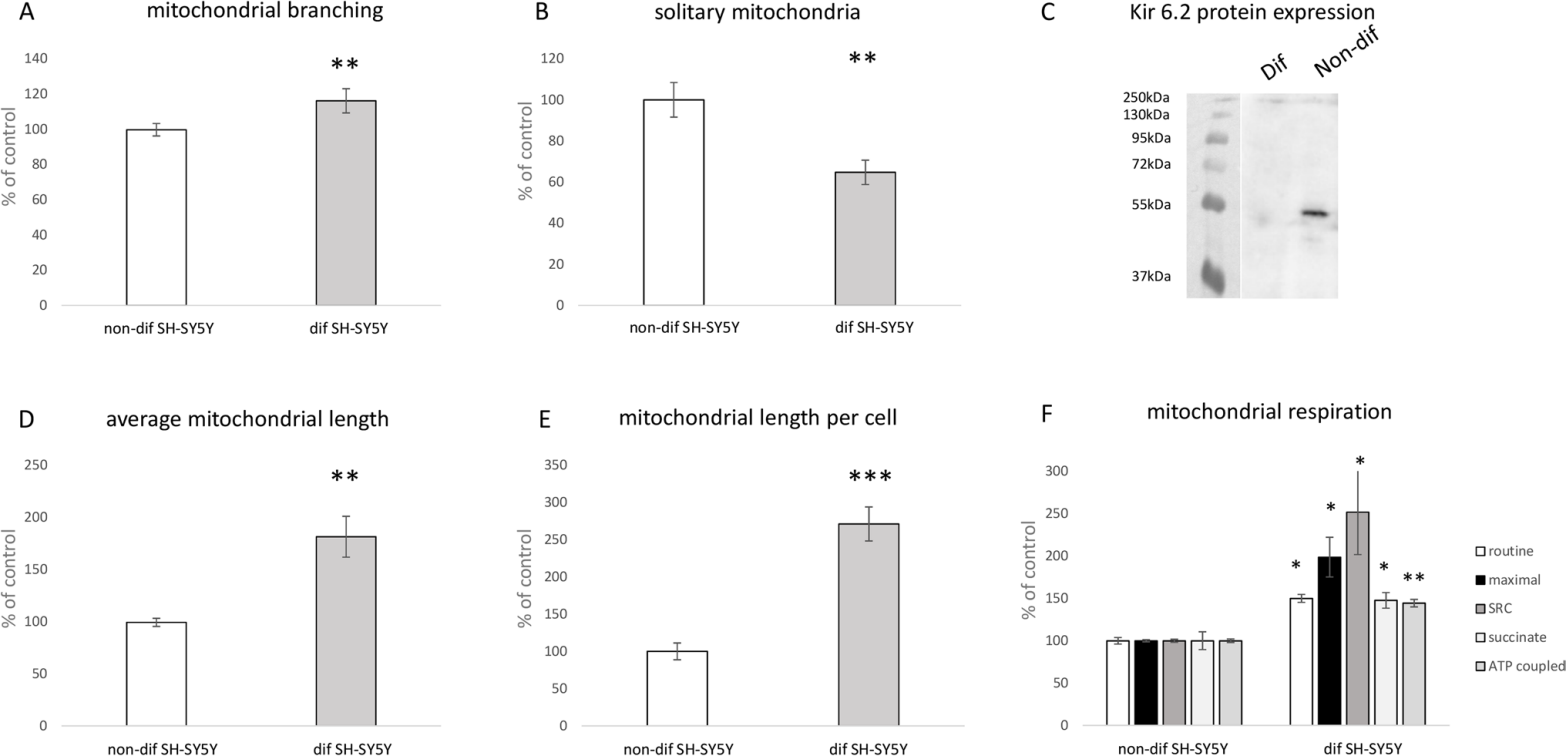
Impact of differentiation process on mitochondrial parameters and Kir 6.2 protein expression. Graphical representation comparing mitochondrial branching (a), solitary mitochondria count (b), average branch length (d) and overall mitochondrial length per cell (e) between differentiated and non-differentiated SH-SY5Y cells. Mitochondrial respiration (f) is characterized by: basal respiration (routine), maximal capacity of electron transport (maximal), spare respiratory capacity (SRC), succinate driven respiratory capacity (succinate), ATP synthesis coupled respiration (ATP coupled). Western blot analysis assessed the expression of Kir 6.2 protein in each cell type (c). Statistical significance between non-differentiated and differentiated SH-SY5Y cells was determined using independent T-test. *p < 0.05, **p < 0.01, **p < 0.001

The primary aim of this study was to assess the regulatory significance of ATP-dependent potassium channels (KATP) in two different types of SH-SY5Y cell lines. We investigated the effects of KATP channel modulation under normal conditions and after inhibiting mitochondrial complex I, a common approach for inducing in vitro Parkinson’s-like neurodegeneration. The initial set of experiments aimed to highlight differences in mitochondrial physiology and morphology between both types of SH-SY5Y cells. Essentially, the mitochondrial network in differentiated SH cells appeared to be more developed compared to those in non-differentiated SH cells. This distinction is graphically represented in Fig. 3 (a, b, d, e), illustrating differences in mitochondrial networks between both cell types. Analysis of mitochondrial length, branching patterns, and overall mitochondrial length in individual cells indicated significantly higher values (p < 0.01-p < 0.001) in differentiated SH-SY5Y cells compared to their non-differentiated counterparts. Furthermore, the fragmentation pattern of the mitochondrial network in differentiated cells was less pronounced, evidenced by a reduced count of solitary mitochondria (p < 0.01) compared to non-differentiated SH-SY5Y controls. All measured parameters of mitochondrial respiration were higher in differentiated cells. In differentiated SH-SY5Y cells, we observed elevated basal respiration and maximal electron transport capacity, spare respiratory capacity (SRC), succinate-driven respiration, as well as ATP synthesis-coupled respiration (Fig. 3f). All Western blot analyses of protein expression revealed a notably larger expression of the KATP subunit Kir6.2, encoded by the KCNJ11 gene, in non-differentiated SH-SY5Y cells (Fig. 3c).

**Fig. 4:**
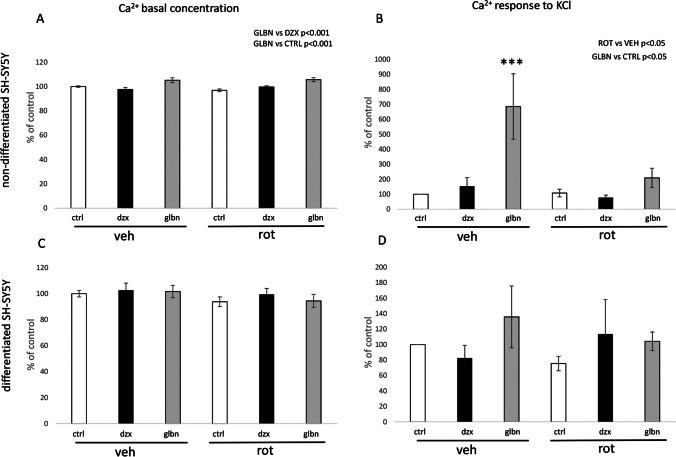
Comparative analysis of Ca^2+^ concentration following 24-hour exposure to KATP modulators under basal conditions and potassium elevation stimulation. The graphical representation illustrates the percentage changes in Ca^2+^ concentration compared to the control group. The effects of KATP modulators, glibenclamide, and diazoxide were examined under both normal and rotenone-induced pathological conditions. To evaluate the factors “rotenone” and “KATP treatment”, two-way ANOVA was employed for statistical analysis. Present statistical significance of main factors is highlighted above the relevant graphs. Significance of experimental groups compared to control cells treated by vehicle identified by Tukey post-hoc test is denoted as *** for p < 0.001

Based on these results, we further examined the potential role of KATP channels through a 24-hour exposure to their antagonists and agonists, namely glibenclamide and diazoxide. These experiments were conducted in both types of SH-SY5Y cells under normal conditions and during pathological processes induced by 50 nM rotenone-mediated complex I inhibition for 24 hours. While our focus was primarily on mitochondrial dynamics, we also investigated some general parameters related to calcium homeostasis and energy metabolism. Initial observations were made regarding changes in basal calcium homeostasis following the 24-hour treatment protocol. As shown in Fig. 4, the statistically significant differences were observed only in the non-differentiated SH-SY5Y cells after glibenclamide treatments (Fig. 4a). Similar effects were observed in measurements of rapid calcium changes triggered by immediate elevation of KCl in both types of SH-SY5Y cells. In non-differentiated cells, glibenclamide exhibited a stimulatory effect on immediate calcium elevation following application of KCl (Fig. 4b). However, the stimulatory effect of glibenclamide was not so robust in non-differentiated cells treated with the rotenone (Fig. 4b). Any of the effects found in non-differentiated cells was not found in differentiated SH-SY5Y cells similarly like after basal calcium concentration evaluation (Fig. 4c, d).

**Fig. 5:**
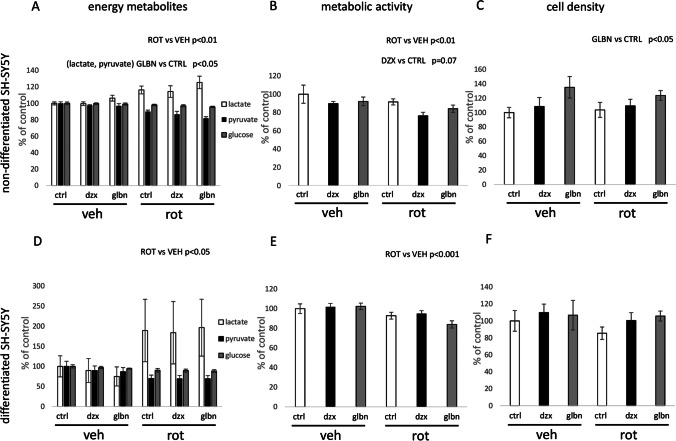
Parametrization of metabolic and mitochondrial impact of KATP modulators in non-differentiated vs. differentiated SH-SY5Y cells. Relative changes are expressed as percentages compared to the control experimental groups. Energy metabolism intermediates were evaluated using NMR analysis (a, e). Metabolic activity was assessed via the MTT test (b, f). Cell density was quantified according to cell counts evaluated using microscopic images (c, f). To evaluate the factors “rotenone” and “KATP treatment”, two-way ANOVA was employed for statistical analysis. Present statistical significance of main factors effects is highlighted above the relevant graphs

**Fig. 6:**
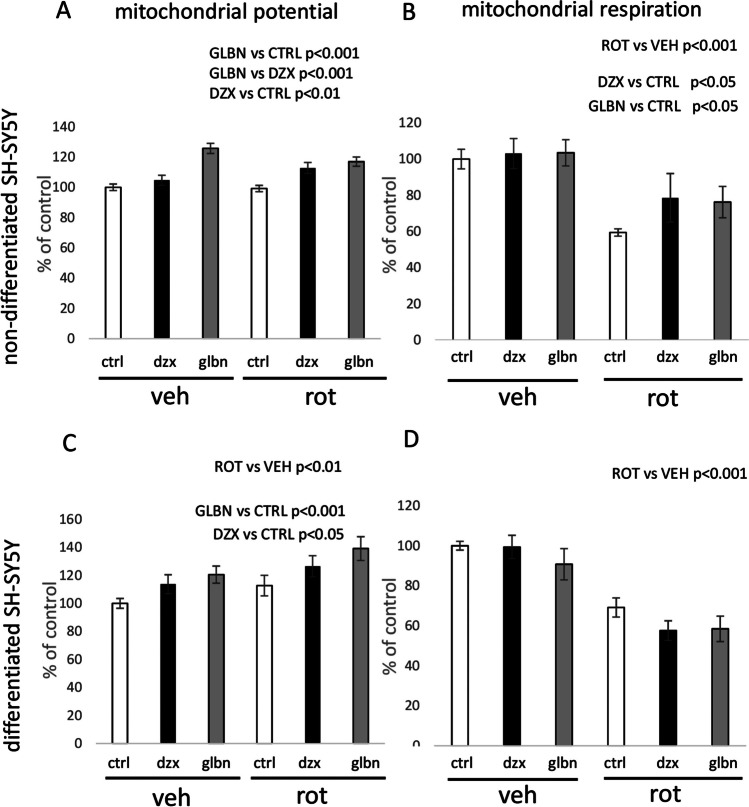
Features of mitochondrial physiology evaluated in non-differentiated vs. differentiated SH-SY5Y cells after exposure to KATP modulators and rotenone for 24 h. The inner mitochondrial potential was quantified indirectly using fluorescence intensity after mitotracker Red FM staining (a, c). Measurements of mitochondrial routine respiration were conducted by measurements of oxygen consumption (b, d). To evaluate the factors “rotenone” and “KATP treatment”, two-way ANOVA was employed for statistical analysis. Present statistical significance of main factors effects is highlighted above the relevant graphs

Subsequent experiments focused on analyzing the metabolic features of our experimental models. A significant effect of rotenone was observed in the elevated lactate concentration in the growth medium, accompanied by a simultaneous lowering in glucose and pyruvate concentrations in both cell types used (Fig. 5a, d). These changes in energy metabolites correlated with a reduction in the basal mitochondrial respiration of both cell types after 24 hours of rotenone exposure (Fig. 6b, d). Finally, the metabolic activity of both types of SH-SY5Y cells was negatively affected by rotenone in both cell types, as demonstrated by the MTT test (Fig. 5b, e), altough 24 hours of rotenone presence in growth medium has stimulatory effect on fluorescence of mitotracker red FM of differentiated cells (Fig. 6c). Anyway, there was not observed any effect of rotenone on cell counts found in both types of SH-SY5Y cells (Fig. 5c, f).

In case of KATP modulators, statistically significant effects of KATP modulators were observed mainly in the case of measurements performed with non-differentiated cells. Inhibition of KATP by glibenclamide resulted in higher lactate concentration in the growth medium of non-differentiated cell cultures (Fig. 5a). The MTT test performed on non-differentiated cells showed only marginally significant toxic effect of diazoxide (Fig. 5b), while no effect on MTT metabolic turnover was observed after 24 hours of glibenclamide presence in growth medium. According to ANOVA, both glibenclamide and diazoxide effectively enhanced mitotracker red FM fluorescence (Fig. 6a) as well as the basal respiration rate in non-differentiated SH-SY5Y cells (Fig.6b), while only effect on mitochondrial potential represented by mitotracker red FM increased intensity remain observable after analysis of differentiated cells (Fig. 6c, d). When comparing the effects of both KATP modulators, permanent blocking of KATP by glibenclamide for 24 hours showed more robust effect on mitotracker red FM fluorescence, as revealed by two way ANOVA. Additionally, microscopy observations provided data about changes in cell population under relevant experimental conditions after KATP treatment. Glibenclamide effectively increased cell counts of non-differentiated cells, while similar effects were absent in differentiated counterparts (Fig. 5c, f).

**Fig. 7:**
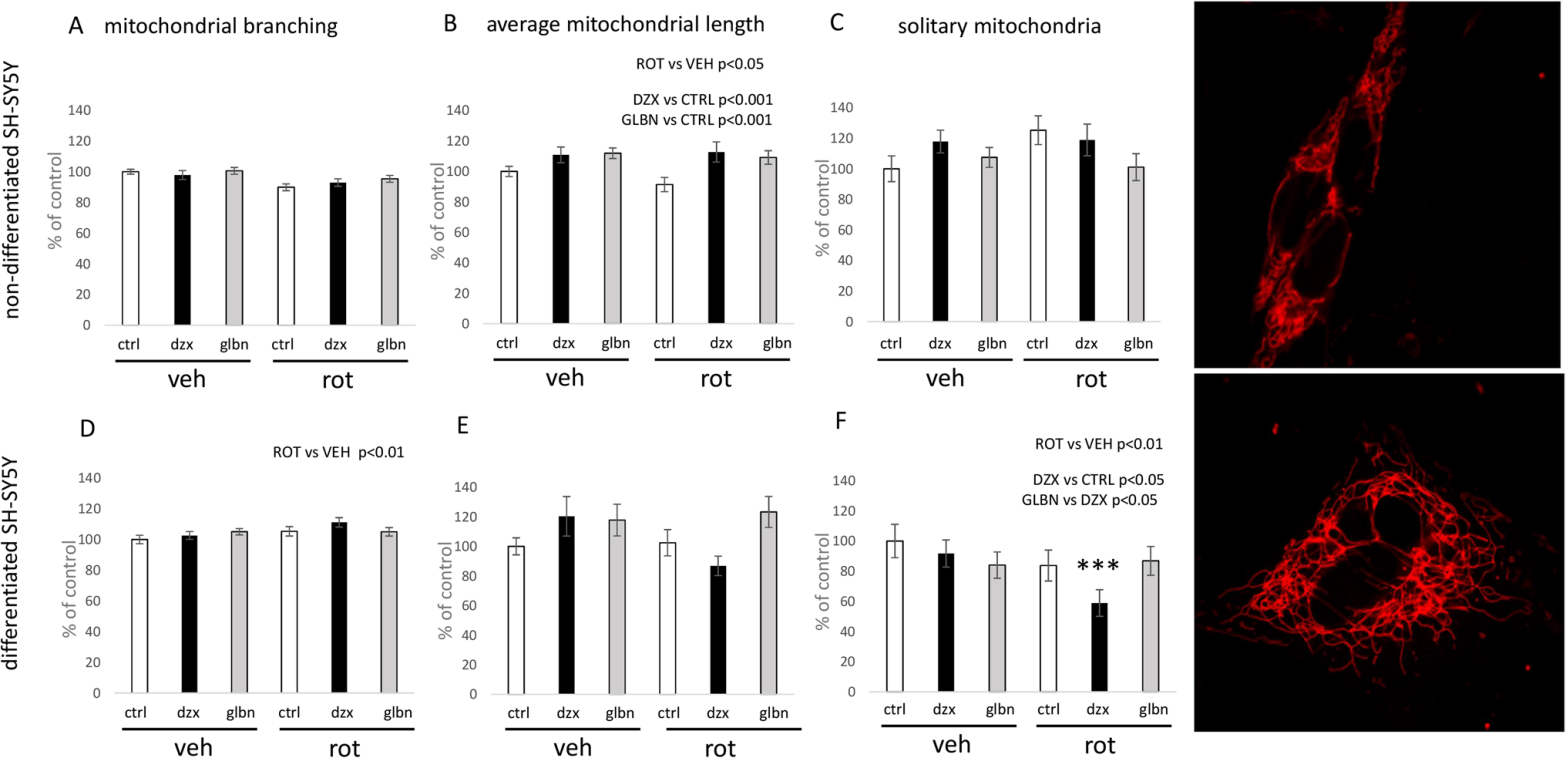
Morphological changes in the mitochondrial network in non-differentiated vs. differentiated SH-SY5Y cells following 24-hour treatment with KATP modulators and rotenone. Graphical illustrations depict morphological alterations in mitochondrial networks, focusing on branching patterns, the proportion of solitary mitochondria per overall count of mitochondrial branches, and the average length of mitochondrial branches in non-differentiated vs. differentiated SH-SY5Y cells. To evaluate the factors “rotenone” and “KATP treatment”, two-way ANOVA was employed for statistical analysis. Present statistical significance of main factors is highlighted above the relevant graphs. Significant change in cells treated with rotenone and diazoxide compared to the control cell culture treated with vehicle was identified by Tukey post-hoc test and is denoted as *** for p < 0.001. Representative images of mitochondrial network observed in both types of the SH-SY5Y cells used for analysis are shown in right raw of the figure

Further analysis focused on identifying changes in mitochondrial morphology in response to the experimental substances used. Evaluation of mitochondrial branching, length, and the abundance of solitary mitochondrial structures revealed different patterns depending on the differentiation status. The effect of rotenone in non-differentiated SH-SY5Y cells was inhibitory, particularly in terms of average mitochondrial length (Fig. 7b). In differentiated SH-SY5Y cells, the same dose of rotenone did not significantly alter the length of mitochondria but had a stimulatory effect on mitochondrial network branching (Fig. 7d). Additionally, the inhibition of mitochondrial complex I led to a significant reduction in the number of solitary mitochondria within the overall mitochondrial networks in differentiated SH-SY5Y cells (Fig. 7f).

In summary, our findings indicate that dysfunction of KATP channel caused by 24 hours exposure to diazoxide as well as glibenclamide has stimulatory effect on mitochondrial length (Fig. 7b). Diazoxide, however, exerts specific effects on mitochondrial network remodeling in differentiated cells. A strong interaction of both used factors (rotenone and KATP treatment) was evident here, as reflected by lowering of the proportion in rod-like mitochondria during KATP opening, with the significance of this effect was observed in higher magnitude in pathological conditions (Fig. 7f).

## Discussion

In this study, we have elucidated the association between the differentiation of SH-SY5Y cells and alterations in the expression of ATP-dependent potassium channels Kir 6.2, shifts in mitochondrial morphology and functions, and variations in cellular metabolism. Furthermore, we have documented the modified responses of both non-differentiated and differentiated SH-SY5Y cells treated with rotenone to the modulators of the functions of ATP-dependent potassium channels. Despite its neuroblastoma origin, the SH-SY5Y cell line has been extensively utilized as a reliable experimental model to the study of Parkinson’s-like neurodegeneration (Ioghen et al. 2023). The impact of ATP-dependent potassium channels (KATP) on neuronal function has been a subject of interest for over three decades, particularly in neurodegenerative disorders such as Parkinson’s disease, ischemic insults, and Alzheimer’s disease (Szeto et al. 2018; Lv et al. 2022). The outcomes of our current study provide valuable insights into the distinctions between dopaminergic progenitor cells and adult dopaminergic neurons derived from the SH-SY5Y cell line, especially in their responses to KATP channel modulators. Given the pivotal role of mitochondrial functions in the mechanism of neurodegeneration, our set of experiments specifically focused on potential alterations in mitochondrial morphology, energy metabolism, metabolic activity, and mitochondrial respiration. The primary objective was to elucidate the characteristic features of both types of the SH-SY5Y cells under normal conditions and during pathological conditions induced by a 24-hour treatment with 50 nM rotenone.

The presented results highlight fundamental differences in mitochondrial network complexity between differentiated and non-differentiated SH-SY5Y cells. Specifically, differentiated cells manifest a significantly more branched mitochondrial network, as indicated by the elevated ratio of junction count per overall mitochondrial branches (Fig. 3a). Furthermore, the mitochondrial network in differentiated cells exhibits reduced fragmentation, as evidenced by a diminished proportion of solitary mitochondrial structures in relation to the overall mitochondrial branches (Fig. 3b). Another distinguishing feature is represented by the enhanced length of individual mitochondrial branches and rod-like structures in differentiated SH-SY5Y cells in comparison to their non-differentiated counterparts (Fig. 3d). In differentiated cells, the mitochondrial network is notably enlarged, accompanied by a substantial increase in the overall mitochondrial length per cell (Fig. 3e). Our findings additionally indicate a increased respiratory capacity in differentiated cells, as evidenced also by a higher rate of mitochondrial oxygen consumption observed through respirometry (Fig. 3f). Notably, our findings concerning mitochondrial length align with a study conducted on mature neurons in mice. In this investigation, damaged mitochondria under ischemic conditions were linked to increased fragmentation and diminished mitochondrial length (Kim et al. 2022). Furthermore, our results are consistent with another study demonstrating that the induction of retinoic acid-mediated differentiation in SH-SY5Y cells resulted in an augmentation of ATP production (Forster et al. 2016).

We characterized the differences in protein expression of the KCNJ11 gene, responsible for encoding Kir 6.2, across the examined cell types. Western blot analysis revealed a nearly exclusive Kir 6.2 expression in non-differentiated SH-SY5Y cells, as depicted in Fig. 3c. The predominant Kir 6.2 expression in non-differentiated cells is notably unexpected, considering the conventional expectation of elevated levels in neuronal structures and other excitable cell types for this type of potassium rectifier (Huang et al. 2007; Fagerberg et al. 2014). Our findings offer pioneering insights, as a lack of relevant assessments regarding Kir6.2 expression changes during differentiation protocols exists in the current literature. Nonetheless, investigations focusing on the rat entorhinal cortex have identified increased expression of this channel subunit in immature neurons during ontogenesis (Lemak et al. 2014).

Subsequent to the characterization of mitochondrial networks and the assessment of selected protein levels, we delved into the effects of KATP modulation using both its agonist (diazoxide) and antagonist (glibenclamide). Remarkably, basal calcium homeostasis exhibited a significant alteration solely in non-differentiated cells, with the most notable distinction observed between the effects of glibenclamide and diazoxide. In comparison to control and diazoxide treated non-differentiated cells, glibenclamide treatment resulted in a noteworthy increase of calcium concentration in control cells as well as in rotenone treated cells, as illustrated in Fig. 4a. These findings align with existing literature, wherein glibenclamide is linked to calcium overload, while diazoxide is acknowledged for its capacity to decrease intracellular calcium levels (Mariot et al. 1998; Sola et al. 2015).

Furthermore, calcium imaging experiments were conducted, revealing no significant effects of 24-hour treatment with KATP modulators or rotenone in differentiated SH-SY5Y cells. Significant alterations in calcium elevation following excitation by potassium chloride were observed after glibenclamide treatment, especially in control conditions in non-differentiated cells, as depicted in Fig. 4b. However, the significance of glibenclamide’s impact was lowered in pathological conditions induced by rotenone presence. Except that, these data indirectly support the notion of increased protein expression of the KCNJ11 gene in non-differentiated SH-SY5Y cells. Notably, the impact of 24-hour glibenclamide treatment on calcium response to KCl in non-differentiated cells represents a novel finding. In the context of the beneficial effects of glibenclamide on motor symptoms during the progression of Parkinson’s disease (Abdelkader et al. 2020), literature search yielded no relevant result on possible change in, for example, dopamine secretory capacity after glibenclamide treatment. Our results, showing elevated calcium response after glibenclamide treatment, suggest that exploring such possibility could be worthwhile in future experiments. Prior investigations have documented the beneficial effects of glibenclamide in various neurodegeneration models, particularly in terms of its ultimate effects on neurons. Several studies have reported an anti-inflammatory effect of glibenclamide in connection with Parkinson’s disease (Abdelkader et al. 2020; Qiu et al. 2021; Ferdowsi et al. 2022). All documented anti-inflammatory effects of glibenclamide are rooted in animal models, highlighting its ultimate impact on neurons. Our results suggest that in vitro prepared adult dopaminergic neurons, characterized by low expression of this type of ion channel, may not be directly influenced by the drug. However, the expression profile of dopaminergic neurons, as well as their supportive neural cells in vivo could be characterized by different features. Additionally, systemic administration of glibenclamide is reported to be ineffective in reaching the cerebrospinal fluid in concentrations necessary for efficacy, necessitating further exploration of mediated effects (Lahmann et al. 2015).

The principal objective of our study was to elucidate the impact of KATP modulation on mitochondrial physiology in two forms of SH-SY5Y cells under both normal and pathological conditions. Subsequent experiments were designed to evaluate the influence of KATP modulation on cell metabolism, with a specific emphasis on mitochondrial metabolism. Analysis of energy metabolism intermediates unveiled a significant increase in lactate production following the inhibition of complex I, concomitant with reduced concentrations of pyruvate and glucose in the growth medium (Fig. 5a, d). Respirometry experiments consistently affirmed the inhibitory effect of rotenone on mitochondrial respiration, evident in a significant decrease in basal oxygen consumption across both studied cell forms after rotenone treatment (Fig. 6b, d). All aforementioned effects align with standard outcomes anticipated following the inhibition of mitochondrial activity in general.

The effects of KATP modulators were predominantly observed in non-differentiated cells, where we documented a significant stimulatory effect of glibenclamide on lactate production, coupled with an increased uptake of pyruvate from the medium (see Fig. 5a). The nature of this phenomenon could be partially explained by the previously observed inhibitory effects of glibenclamide on mitochondrial activity. Data from several studies suggest that the effects of glibenclamide on mitochondrial metabolism are positively correlated with those of metformin (Salani et al. 2017). Besides mitochondrial parameters, the overall metabolic activity, as assessed through MTT tests, unveiled only a marginal toxic effect of diazoxide (diazoxide p = 0.07) in non-differentiated cells, but not in differentiated SH-SY5Y cells (Fig. 5b). On the other hand, glibenclamide effect was characterized as a beneficial for the cell count in non-differentiated SH-SY5Y (Fig. 5c). On the basis of previous studies, it is evident that the effects of both KATP modulators strongly depend on the chosen experimental model. The impact of both KATP modulators on cell viability was documented by Yilmaz et al. (2015), where diazoxide treatment at a concentration of 10 μM was identified as beneficial for renal tubular cells (Yilmaz et al. 2015). In the same study, glibenclamide exerted toxic effects on the renal cells at the same concentration and time of treatment. The neuroblastoma cells used in our experiments evidently exhibit different sensitivity to the studied KATP modulators. A study published by Du et al. (2016) documented the protective effect of glibenclamide against harmful ferrous influx into the SK-N-SH cells, which constitute the parental cell line for the SH-SY5Y subline (Du et al. 2016). Diazoxide was identified as a negative agent in the chosen experimental procedure. Our experiments with differentiated SH-SY5Y cells further revealed only the toxic effect of rotenone. No significant effects of diazoxide or glibenclamide on the cell viability were observed in differentiated cells (Fig. 5e, f). This phenomenon could be attributed to the lower expression of Kir 6.2 in differentiated cells, as mentioned earlier.

Significant effects of glibenclamide and diazoxide were evident in alterations of mitochondrial potential and respiration in non-differentiated cells, with both KATP modulators displaying a stimulatory effect following 24-hour exposure. In the case of differentiated SH-SY5Y cells, only glibenclamide exhibited a significant stimulatory effect on mitochondrial potential after the 24-hour treatment (Fig. 6c). The observed effect of diazoxide (Fig. 6a) is partially consistent with previous studies, as the opening of mitochondrial KATP channels has stimulating effects on mitochondrial respiration, as evidenced by oxygen consumption (Akopova et al. 2020).

The situation with glibenclamide is intriguing, as earlier investigations have reported inhibitory effects on mitochondrial respiration upon the blockade of mitochondrial KATP channels (Fernandes et al. 2004; Engbersen et al. 2005; Salani et al. 2017). However, other studies have documented stimulatory effects of KATP blockers on mitochondrial respiration under different experimental conditions (Skalska et al. 2005). Notably, our experiments differed from previous studies in terms of duration, as most cell-based experiments have primarily focused on immediate or short-term effects of KATP modulators on mitochondrial respiration. In contrast, our experiments yielded results after a 24-hour treatment with both KATP modulators of both forms of SH-SY5Y cells. This prolonged exposure more closely resembles clinical conditions of long-term treatment, which can be interpreted as a chronic alteration of KATP channel function.

The observed stimulatory effect of rotenone treatment on mitochondrial potential in differentiated cells (Fig. 6c) represents an unusual finding in our experiments. It is essential to highlight that the method employed for measuring mitochondrial potential is based on mitotracker Red FM fluorescence quantification. Rotenone is acknowledged as a potent stimulator of reactive oxygen species (ROS) production, while mitotracker Red FM is a probe known for its high affinity for ROS (Buckman et al. 2001). Considering the noted decrease in mitochondrial respiration in both types of SH-SY5Y cells, this phenomenon may be linked to an increased ROS generation induced by rotenone in differentiated cells.

The comprehensive analysis of morphological alterations in mitochondrial networks under diverse experimental conditions constitutes a significant contribution of our study. The implication of KATP channels in mitochondrial dynamics is a well-recognized phenomenon, and their role in reshaping the mitochondrial network has been previously elucidated by Peng et al. (2018) in a cellular model based on rat adrenal PC12 cells subjected to Parkinson’s-like neurodegeneration. In their investigation, the authors observed negative effects of the combination of an agonist with rotenone on mitochondrial footprint in PC12 cells, along with an increased fragmentation pattern in the mitochondrial network (Peng et al. 2018). These results were positively correlated with behavioral tests in a parallel animal study, and the effect of the selective mitochondrial KATP blocker 5-hydroxydecanoate was found to be slightly beneficial.

Our findings highlight critical differences between the chosen cell types, as evidenced by the protein expression of KCNJ11 between differentiated and non-differentiated SH-SY5Y cells. Non-differentiated SH-SY5Y cells, characterized by higher KCNJ11 expression, were observed to be more sensitive to the KATP modulators used in our study. The effect of both opening and blocking KATP channels was evidently beneficial for the average branch length of mitochondria in non-differentiated cells (Fig. 7b).

Rotenone treatment had a statistically significant downregulating effect on mitochondrial length in neuroblastoma progenitor cells (Fig. 7b). Focusing on differentiated cells, mitochondrial fragmentation was significantly influenced by rotenone, resulting in a lower number of solitary mitochondria per overall mitochondrial network (Fig. 7f). Rotenone also exhibited a stimulatory effect on mitochondrial network complexity (branching) (Fig. 7d). This observation may be correlated with a previous study wherein the dissipation of mitochondrial potential in non-differentiated SH-SY5Y and BJ fibroblast cells by FCCP for 3 hours resulted in a significant decrease in the average length of mitochondrial branches or rods (Valente et al. 2017; Bakare et al. 2021). In contrast to studies with FCCP on SH-SY5Y and BJ fibroblast cells, our 24-hour rotenone treatment did not lead to a significant increase in the count of rod-like mitochondria, which would indicate fragmentation.

Based on documented evidence, the most interesting finding is the stimulatory effect of diazoxide treatment on the count of sole mitochondria in differentiated cells. This phenomenon is especially pronounced after the induction of pathological conditions, where it appears to synergize with the reducing effect of rotenone on the number of solitary mitochondria rods (Fig. 7f). This finding is somewhat surprising, as the downregulation of solitary mitochondria is typically considered a positive event (Valente et al. 2017). However, based on relevant data from other authors, studies using 50 nM concentrations of rotenone on differentiated cells seem to develop pathological changes over several days, impacting cell viability and mitochondrial movement across SH-SY5Y cells (Borland et al. 2008). The concentration of rotenone used in our study can therefore be considered mild. Compensatory adaptation events occurring in living cells during exposure to rotenone could provide a hypothetical explanation for our observations, but this requires further investigation. More interestingly, the observed effect of diazoxide is the only and highly specific effect of KATP modulators-agonist on mitochondrial network remodeling observed in differentiated cells. With this in mind, we need to consider different effectors of diazoxide actions, especially since Kir 6.2 concentration is diminished in this type of SH-SY5Y. In light of recently documented diazoxide-sensitive mitochondrial KATP coded by CCDC51 gene (Paggio et al. 2019), we should take into account the possible role of this recently described potassium channel in the observed changes.

In conclusion, our data, when contextualized with existing findings, underscore the model-dependent nature of KATP channel effects. While the majority of the observed effects following treatment with KATP modulators favors the increased sensitivity of non-differentiated cells, distinctive responses are evident in differentiated SH-SY5Y cells when exposed to a KATP agonist. Despite the subdued protein expression of Kir 6.2 in differentiated cells, we noted alterations in the number of solitary mitochondria and mitochondrial potential in response to KATP modulator treatment in differentiated SH-SY5Y cells. This phenomenon warrants further investigation in future experiments and highlights the limitations of our study, particularly in the need to correlate the observed data with relevance of specific effects of intracellular KATP according to their localization. Therefore, future experiments should include a wider variety of KATP modulators to address the question of distinct effect of mitochondrial vs membrane KATP. Different differentiation protocols of SH-SY5Y as well as possible isolation of dopaminergic neurons from in vivo experiments should be performed to test potential Kir 6.2/6.1 expression under different conditions. Despite these limitations, our data provide additional support for the potential therapeutic efficacy of ATP-gated ion channels and their modulators in addressing neurodegeneration stemming from mitochondrial dysfunction. This is notably apparent in the modulation of mitochondrial morphology and concomitant alterations in energy metabolism of non-differentiated SH-SY5Y cells expressing Kir 6.2. The suitability of KATP modulators as adjunctive treatments for neurodegenerative disorders will necessitate case-specific assessments according to the unique etiopathogenesis of individual cases of neurodegeneration.
